# Book Review

**DOI:** 10.1120/jacmp.v7i3.2299

**Published:** 2006-08-24

**Authors:** James. B. Smathers

**Affiliations:** ^1^ Professor Emeritus Department of Radiation Oncology UCLA B265 200 UCLA Medical Plaza Los Angeles Ca. 90095‐6951

## Abstract

*Structural Shielding Design and Evaluation for Megavoltage X‐ and Gamma Ray Radiotherapy Facilities; National Council on Radiation Protection and Measurements* (NCRP Report 151); Issued December 2005, ISBN‐13: 987‐0‐29600‐87‐1; Price $100 USD (hard copy), $80 (down loadable PDF), $170 (combined package)

This report has been long awaited by the therapy community and it serves at least two distinct communities of physicists; those newly entering the field that do not have a library shelf full of previous NCRP reports and the other group are the more experienced physicists that have all of the previous reports.

For those just entering the field, this is a must buy book if you intend to do therapy shielding design consulting or just wish to be brought up to date on the current techniques in the field of shielding calculation. I give that strong an endorsement to the report as I believe the regulatory agencies will be using it determine if the calculations you submit for approval have been done in a proper manner. To be sure there are other techniques and equations that one can use but the burden of proof will be on the physicist to show that the non‐NCRP approach is equally valid. Life is too short to have to go through that with regulators.

Now for the readers that have the old reports, the question is whether this report is worth the money or can I still get buy with the old reports. Regrettably, I think one will have to buy it. I reach that conclusion after reviewing the old reports on my shelf and comparing topic coverage in report 151 with the previous reports by the NCRP.

For both groups let me do a quick survey of the report with an emphasis where new information is clearly included and useful. The Introduction and Calculational Methods, sections 1 & 2, clearly present the equations which they recommend and define all of the symbols used in a clear manner. The equations are not derived but rather references are given to the appropriate paper or book. In chapter 3, the workload, use factor, and absorbed dose rate considerations are discussed. There is new information here which includes workload factors vs. gantry angle reflecting more current accelerator usage. Also occupancy factors are presented which are lower than some used in the past. The special considerations that TBI irradiations, IMRT treatments require are also discussed and recommendations are given for how to properly treat the typically higher doses involve.

The photo‐neutron problem is clearly presented and more calculational approaches summarized which should be of great help to someone evaluating the shielding of a high energy accelerator. For those who do not have a copy of NCRP 79, there is no need to find one. The treatment of neutrons in NCRP 151 surpasses that of the old NCRP 79.

The therapy isotopes appear to have fallen in the crack. The nice curves of the attenuation of Ir‐192, Cs‐137, and Co‐60 which are in NCRP 49 are not in the Appendices of NCRP 151. For those doing HDR vault designs, the data in NCRP 49 will still be of some value.

Section 4 deals with Structural Details and it is here in this chapter that the prescriptive flavor of the report is lost. Do not throw out your NCRP 49. The clear recommendations for duct wrapping are given in NCRP 49 not in this section. Nor are the recommendations on gap width, sliding door overlap distances as given in NCRP 51 included.

Section 5 treats “sky shine” in a very clear and direct manner. Most useful to this reviewer was the inclusion of a comparison of calculated dose vs. distance from the facility with actual measurement data. The uncertainties involved in this type of calculation are clearly illustrated. It is in this section that the special accelerator devices such as Tomotherapy, and Robotic Arm, and the old but proven Cobalt units are discussed. The special shielding considerations of each are discussed and reference made as to how to use/modify the equations previously given to properly calculate the shields for these systems.

Section 6 gives a few suggestions on Shielding Evaluation Surveys but the strength of the book is the very detailed set of calculation examples in Section 7. This section does a dual energy accelerator with neutron considerations in full detail as well as the very special room design for a robotic arm system. If the reader has a shielding calculational system this would be a great example to bench mark it against. For those with no such system, just follow the example as you set up your spread sheets to do all of the required calculations.

The three appendices include the following:

- Two pages of attenuation curves vs. energy for various materials.
- Fifteen pages of shielding tenth values vs. energy and materials, wall reflection coefficients, and accelerator radiation characteristics.
- Twenty two pages on neutron measurement techniques.

All of them contain useful information and the information contained in Appendix A & B appears to have been selected to allow one to calculate the example problem of section 7.

In the preface the NCRP acknowledges the participation of the American Association of Physicists in Medicine (AAPM) in the writing of this report. In recognition of the AAPM's contributions to the project, A 20 % discount is available to American Association of Physicists in Medicine members for all online purchases by entering the code aapm49151 at checkout. For additional information contact David A. Schauer, ScD, CHP, Executive Director, at schauer@NCRPonline.org, 301.657.2652 (x20) or 301.907.8768 (fax)

All in all a nice document that will be used in shielding calculations for a number of years.

NCRP # 49 Structural Shielding Design and Evaluation for Medical Use of X Rays and Gamma Rays of Energies Up to 10 MeV, Issued September 1976

NCRP # 51 Radiation Protection Design Guidelines For 0.1 – 100 MeV Particle Accelerator Facilities, March 1977

NCRP # 79 Neutron contamination From Medical Electron Accelerators, Issued November 1984

